# Perceptions and moral distress in surplus embryo disposition among Japanese IVF patients: a qualitative study

**DOI:** 10.3389/frph.2025.1646340

**Published:** 2025-09-04

**Authors:** Shizuka Katsuzaki, Yoshiyuki Takimoto, Chikako Morioka, Masahiro Nakayama, Tatsuya Harada, Yoko Urata, Tomonori Ishikawa, Masatoshi Takagi

**Affiliations:** ^1^ Patient Relations and Clinical Ethics Center, The University of Tokyo Hospital, Tokyo, Japan; ^2^ Department of Pediatrics and Developmental Biology, Institute of Science Tokyo, Tokyo, Japan; ^3^ Department of Patient Safety and Quality Management, Kobe University Hospital, Hyogo, Japan; ^4^ ASKA Ladies Clinic, Nara, Japan; ^5^ Medical Park Bayfront Yokohama, Kanagawa, Japan; ^6^ Division of Reproductive Medicine, National Center for Child Health and Development, Tokyo, Japan; ^7^ Center for Reproductive Medicine, Institute of Science Tokyo Hospital, Tokyo, Japan

**Keywords:** embryo disposition, moral distress, infertility treatment, qualitative research, Japan

## Abstract

**Background:**

The growing use of *in vitro* fertilization (IVF) in Japan has led to an increase in surplus frozen embryos and the associated ethical dilemmas. Cultural values, institutional practices, and limited legal guidance contribute to patient distress and uncertainty. However, little is known about how Japanese patients perceive surplus embryos, and how these views relate to their emotional experiences and support preferences.

**Methods:**

This qualitative study included semi-structured interviews with 46 female patients who had undergone IVF recruited from three Japanese fertility clinics with varying policies for embryo disposition. The interviews were conducted online between May 2024 and March 2025. Data were analyzed using thematic analysis guided by Boyatzis's inductive coding method.

**Results:**

Participants conceptualized embryos in three main ways: as “life,” as genetically connected “blood ties,” or as “cells or eggs.” These perceptions—now more diverse than in earlier reports—influence emotional responses and dispositional decisions. Viewing embryos as life forms is often associated with guilt and sorrow, particularly in the absence of clear information or acceptable options, and could be understood as a form of moral distress from the patient's perspective. Practical considerations, such as storage fees and future family planning, also shaped both perceptions and emotional burden. Gendered responses were observed: women with female-factor infertility tended to internalize blame, while male-factor diagnoses allowed for emotional distancing. Institutional factors, including storage fees, a lack of legal frameworks, and stigma, shape moral distress and decision-making. While embryo donation for research is often preferred, donation to other patients is strongly opposed, reflecting cultural concerns about kinship and genetic identity. Support preferences range from symbolic closures to informal peer dialogues.

**Conclusion:**

Patient perceptions of surplus embryos are shaped by cultural values, gendered expectations, and institutional factors, and practical constraints. These findings highlight the need for culturally responsive, ethically grounded, and flexible care models that acknowledge moral distress as experienced by patients and the growing diversity in how embryos are conceptualized.

## 1 Introduction

In Japan, the use of assisted reproductive technology (ART) has steadily increased, with approximately 10% of all births in 2022 resulting from *in vitro* fertilization (IVF) (1). In the same year, ART became eligible for public health insurance, thereby expanding access to treatment. A typical IVF protocol involves the fertilizing of multiple eggs and the freezing of surplus embryos for future use. Patients were periodically asked to decide whether to continue storing, discarding, or donating embryos for research.

Although IVF has helped many patients achieve pregnancy, it also entails physical, emotional, and financial burdens (2). Decisions regarding surplus embryos are fraught and often accompanied by ethical dilemmas and emotional distress (3).

Some patients report distress, saying, for example, “I feel like they are life,” or “If I must discard them, I would rather have them returned to my body.” In addition, clinics in Japan have responded by offering symbolic options, including memorial services (4) and even the return of preserved embryos (5). These practices reflect how emotionally charged and socially visible the issue has become in Japan, frequently appearing in the public discourse and patient communities.

International studies have shown that patients' perceptions of embryos, such as viewing them as “potential persons” or “children,” can significantly influence disposition decisions due to the emotional weight they carry (6, 7). However, how these perceptions are shaped by Japan's cultural and institutional contexts remains poorly understood. Compared to Western countries, Japan lacks clear national guidelines for embryo donation and disposal. Previous research has suggested that Asian patients may be less willing to donate embryos, potentially because of their culturally embedded values (8). In East Asian societies, including Japan, reproductive roles are often closely tied to identity and social position, making decisions regarding embryo disposition particularly sensitive (9).

In this study, the term “moral distress” is used in an expanded sense to describe the emotional and moral conflict experienced by patients when they are unable to act in accordance with their values due to institutional constraints or limited available options. While the term has traditionally been applied to healthcare professionals (10), some recent literature has explored its broader application (11, 12). We employ the term here with interpretive intent, to capture the intensity and complexity of patients' emotional struggles in the context of embryo disposition decisions.

Despite the increasing number of ART users in Japan, few qualitative studies have examined patients' lived experiences and support needs related to embryo disposition. Understanding how Japanese patients perceive surplus embryos—and how these views relate to moral distress and help – seeking —can guide the development of more culturally sensitive reproductive care.

## 2 Materials and methods

### 2.1 Study design

This study employed a qualitative design using semi-structured interviews to explore how patients who underwent IVF in Japan perceived surplus frozen embryos, and how these perceptions influenced decision-making and experiences of emotional and moral conflict, hereafter referred to as “moral distress” as defined in the Introduction.

### 2.2 Participants and recruitment

The participants were Japanese women who had undergone IVF and had experience with embryo cryopreservation. Individuals with severe psychiatric conditions or deemed unsuitable for participation by a physician were excluded. A purposive sampling strategy was used to recruit participants with the relevant experience. Recruitment was conducted across four medical institutions selected to ensure diversity in clinical practices and accessibility, including a university hospital, a national hospital, and two IVF clinics with differing policies on embryo donation and memorial services. Participants resided in or around the metropolitan areas of Tokyo, Kanagawa, and Nara. Participation was voluntary, and all participants provided informed consent.

### 2.3 Data collection

Semi-structured one-on-one interviews were conducted by the first author with 46 participants between May 2024 and March 2025. All interviews were conducted online as preferred by all participants. The interviews lasted between 45 and 90 min and followed a predeveloped interview guide (Table 1). The interview guide was developed by the first author by referring previous studies and refined in consultation with the co-authors.

**Table 1:** Interview guide.

Section	Sample questions
1. Experiences with infertility treatment and embryo freezing	- Could you tell me about your experience with infertility treatment? - What was it like when your embryos were frozen for the first time?
2. Perceptions of frozen embryos	- How do you perceive the embryos that are currently in storage? - Has your view of them changed over time? If so, how? - Are there any metaphors, images, or expressions that come to mind when you think of them?
3. Thoughts and decisions about embryo disposition	- Have you made any decisions about what to do with the embryos? - If yes, what led to that decision? If not, what are you currently thinking? - Have you discussed this with others (e.g., your partner, family, medical staff)?
4. Considerations beyond storage or discarding	- Apart from storing or discarding them, have you ever wished for any other options? - Have you heard of options like memorial disposal or compassionate transfer? How did you feel about them?
5. Influences and reflections	- Are there any particular experiences, conversations, or social factors that influenced how you see your embryos? - Have you ever heard how others think about their embryos? Did that affect your own thoughts?
6. Support needs and communication preferences	- Have you ever wanted someone to talk to about these decisions? - What kind of support or information might have helped you? - How do you feel about counseling?
7. Motivation for participating in this interview	- What made you decide to take part in this interview?

### 2.4 Data analysis

Interview transcripts were analyzed thematically using Boyatzis' inductive method (13). Transcripts were reviewed line by line, and the relevant segments were coded using NVivo 15 software. To ensure analytical clarity and consistency, two separate codebooks were developed—one focusing on participants' perceptions of embryos (e.g., as “life,” “genetic,” or “cells”) and the other on the presence or absence of emotional burden related to embryo disposition.

Themes were identified by examining the intersections between these two axes and grouping similar codes into conceptual categories. These categories were further refined and organized into six overarching themes through focused comparison and content-based classification. Although this method did not require theoretical saturation, no substantial new themes emerged after approximately 30 interviews, indicating that conceptual saturation was achieved. The remaining interviews helped to refine and deepen the existing themes. The coding was reviewed after 1 month for internal consistency. Professionals in psychosomatic medicine, pediatrics, obstetrics and gynecology, nursing, and midwifery provided feedback on the coding process.

### 2.5 Ethical approval

This study was conducted as part of a multi-institutional project and approved by the Research Ethics Committee of the University of Tokyo Faculty of Medicine (Approval No. 2023247NI). This report presents the qualitative components of a broader mixed-methods study. All participants received verbal and written explanations of the study and provided written informed consent. The study was conducted following the Japanese “Ethical Guidelines for Life Sciences and Medical Research Involving Human Subjects” (14) and the Declaration of Helsinki.

## 3 Results

### 3.1 Participants' backgrounds

Between May 2024 and March 2025, 46 participants were recruited from four institutions through posters, outpatient explanations, and postal invitations. Although recruitment was conducted at four sites, participants were ultimately enrolled from three of these institutions, with the majority recruited from a clinic that charged approximately ¥50,000 per year for embryo storage, which was often paid out-of-pocket and relatively high compared to other participating institutions. Most participants were in their 30s to 40s, had undergone a single egg retrieval cycle, and became pregnant after one or two embryo transfers (Table 2).

**Table 2:** Participant characteristics (*n* = 46).

Variable	*n* (%)	Mean (range)
Participating institutions		
A	35 (76.1)	
B	3 (6.5)	
C	8 (17.4)	
Age (years)		35.3 (27–45)
Duration of treatment (years)		3.6 (0–15)
Number of egg retrievals		1.3 (1–4)
Number of frozen embryos		5.9 (2–13)
Number of embryo transfers		2.3 (0–10)
Number of surplus embryos		3.5 (1–10)
Number of children		
0	8 (17.4)	
1	27 (58.7)	
2	11 (23.9)	
History of miscarriage		
None	29 (63.0)	
Once	11 (23.9)	
Twice	3 (6.5)	
Three times	3 (6.5)	
Desire for a second child		
Yes	20 (43.5)	
No	16 (34.8)	
Unsure	10 (21.7)	
Cause of infertility		
Female factor	14 (30.4)	
Male factor	8 (17.4)	
Both	1 (2.2)	
Unexplained	23 (50.0)	
Disposition choice		
Extended cryopreservation	37 (80.4)	
Discarded	8 (17.4)	
Undecided	1 (2.2)	
Employment status		
Employed	36 (78.3)	
Unemployed	10 (21.7)	

Percentages may not total 100% due to rounding. Institutions A–C are anonymized labels used for descriptive purposes only and do not correspond to actual facility names.

### 3.2 Results of thematic analysis

Thematic analysis identified six key themes (Table 3), each addressing distinct aspects of how participants perceived surplus embryos and approached disposition decisions. In addition to the six themes identified through thematic analysis, some topics were mentioned frequently across interviews, but were not presented as separate themes because of their overlap with multiple categories. Many participants stated that they had not told their parents about ART. Others mentioned obtaining information or exchanging experiences regarding infertility treatment through social media. These topics were observed across different thematic contexts and were, therefore, not isolated as distinct themes. To provide a clearer overview of the distribution of perspectives while preserving the qualitative nature of our analysis, we created an additional Supplementary Table S1. Supplementary Table S1 outlines the approximate number of participants associated with each thematic category, complementing Table 3, which focuses on illustrative quotes.

**Table 3:** Identified themes and representative quotations from participants (original Japanese with English translation).

Theme	Subcategory/label	Example narratives
(1) Perceptions of surplus embryos	Perceiving embryos as life	“The fact that it has fertilized and is undergoing cell division means that life has already begun.” (14) “After giving birth to my first child, I started to feel like there were still two lives remaining.” (28)
	Perceiving embryos as genetically related	
	Connection to oneself	“I do feel like I’m the mother.” (33) “I don't even know if it would implant, but it kind of feels like my child.” (40)
	Connection to the born child	“My husband and I always refer to them as siblings.” (2) “When we started thinking about a second child, I realized—yes, they are siblings.” (5)
	Perceiving embryos as eggs or cells	“It doesn't feel like a life yet. Honestly, it just feels like an egg, or like a cell.” (7) “It still feels like one of those cells you see in science class.” (43)
(2) Decisions on embryo disposition	Choosing to discard	
	Gave up on having a second child	“Instead of making another child and spreading myself thin, I really wanted to devote all my attention to this child.” (15) “I used to think my first child would be lonely without a sibling, but over time, I began to feel that I was physically too old to go through with it.” (53)
	Financial burden	“It costs quite a bit, and given my age, there's the issue of whether I could even get pregnant again if we transferred another embryo.” (17) “The high storage fee is a major burden, so we’re considering discarding the embryos this time.” (24)
	Choosing to extend storage	
	Embryos were considered too important to discard	“Choosing the path of discarding it ourselves—it kind of felt like we were killing it.” (37) “I just didn't want to think about the idea of throwing life away.” (48)
	Not wanting treatment efforts to go to waste	“There was someone at my workplace who went through infertility treatment and discarded their embryos. Two or three years later, they said they regretted not keeping them.” (21) “The egg retrieval was really painful, and I knew right then I didn't want to go through it a second time.” (34)
	Postponing the decision for now	“The reason we extended storage recently is that we haven't yet decided whether to try for a third child.” (26) “I couldn't even consider more treatment, and if we discarded them, it would all be over. So, I just thought we’d extend for now.” (29)
(3) Attitudes toward embryo donation	Donation for research	“But if I imagine it being torn apart or subjected to harsh conditions, I’d rather choose disposal.” (16) “If the embryos contributed to medical progress—if they had some kind of meaning—it would be comforting to me.” (26)
	Donation to other patients	“If that child showed up in front of me one day, I don't think I could turn them away, so I’d rather not get involved in something so complicated.” (12) “I’d feel sorry if the child ever wanted to find their biological parents.” (28) “Our embryos are our own, connected to our blood—we want them returned to us. We see them as personal property.” (38)
(4) Views on ceremonial practices	Desire for a memorial	“If the clinic said, ‘We will offer a memorial if you choose disposal,’ I think the emotional hurdle would be lower.” (31) “I looked into mizuko kuyo out of curiosity, but some articles said it was just a money-making scheme, so in the end I didn't do it.” (33)
	Discomfort with taking the embryo home	“If I had the embryo with me, I probably wouldn't be able to go through with discarding it. It would feel too sad.” (41) "I can understand why someone who couldn't have a child might want them, but honestly, I’m totally fine with having them discarded." (52)
	Reactions to compassionate transfer	“To me, it's just a matter of whether you dispose of it outside the body or inside the body.” (4) “It would definitely place some burden on my body. I personally wouldn't do it.” (7) "It sounds really irresponsible, but I hated the idea of discarding them, and at one point I even thought about just putting them back without really preparing for anything."(50)
(5) Infertility and self-perception	Impact of infertility attribution on perceptions and emotions	“When we were tested as a couple, everything was fine on my end, but there were issues on my husband's side.” (21) “I have elevated antiphospholipid antibody levels.” (35) “No one said it directly, but I personally assumed it was a pickup disorder or a problem with my fallopian tubes.” (20)
	Identifying as infertile	“There's a part of me that feels like I’m lacking something—that not being able to conceive in the normal way makes me feel somehow inadequate.” (16) “Now I just think, well, it can't be helped—I’m this age.” (25)
	Jealousy toward others	“Most of my friends have kids, so when we meet up, they naturally talk about children. I know it can't be helped, but it was hard for me.” (32) “They conceived naturally, and everything went smoothly. Meanwhile, I’m left wondering, ‘Why can't I get pregnant naturally? Why is this so painful for me?’” (34)
(6) Support needs	Interest in counselling	“I fully understand that the decision is mine to make, but I think I need to talk to someone to sort out my feelings.” (26) “I wish I could talk more casually about it—whether at the clinic or elsewhere.” (29) “Maybe going through counseling would change how I feel, but I still wonder if I could really go through with discarding them.” (42)
	Reasons for interview participation	“I didn't know much about infertility treatment myself, so if there's research in that area, I’d like to support it.” (10) “I felt like it was finally the right time to talk about it—maybe because I had a child.” (22)

All participant quotations were originally spoken in Japanese and translated into English by the research team. Participant ID numbers are non-sequential, as some individuals consented to participate but did not proceed to interview.

### 3.3 Theme 1: perceptions of surplus embryos

**Figure 1:**
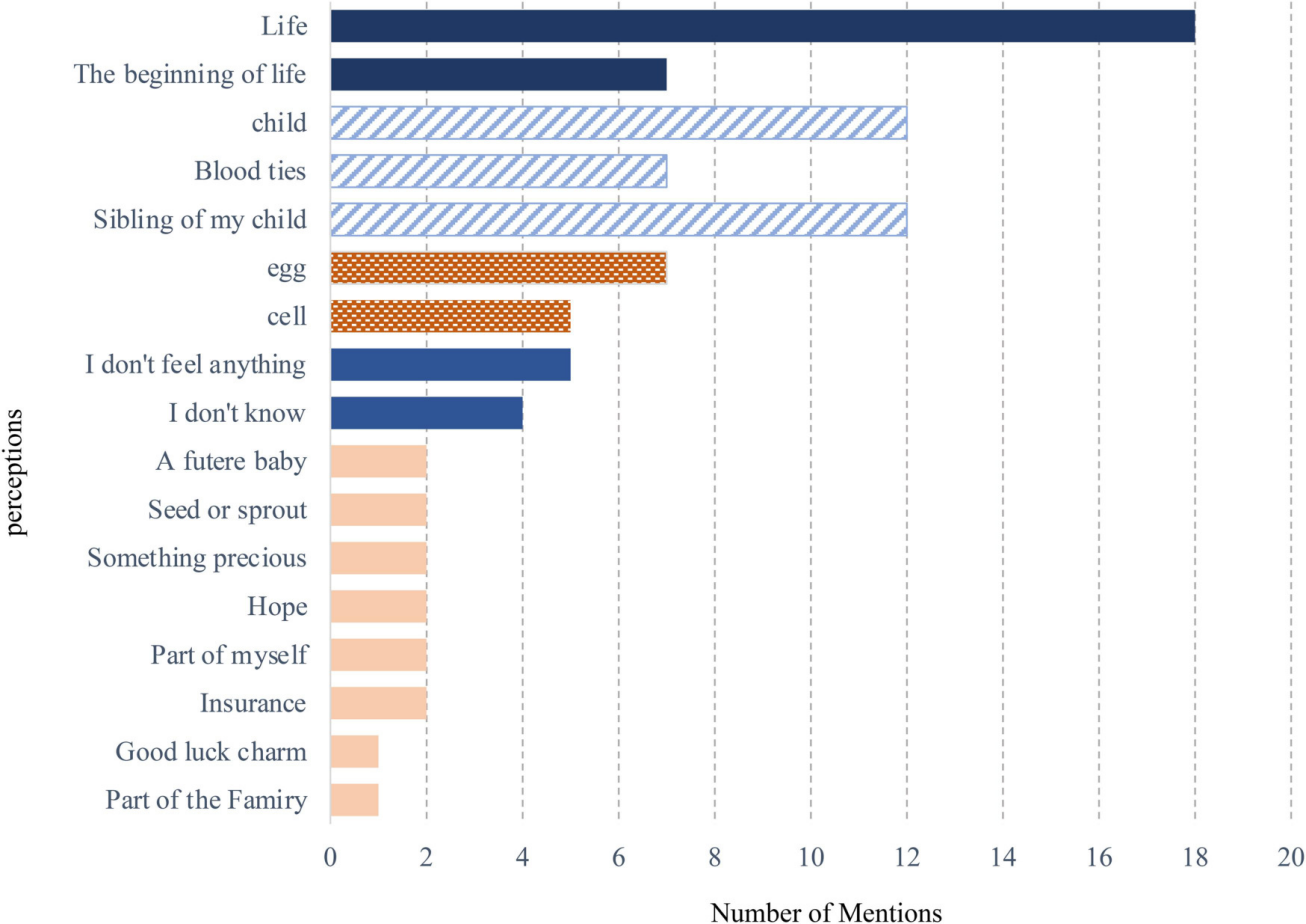
Participants’ perceptions of surplus embryos and the frequency of mentions (*N* = 46; multiple responses allowed). This figure presents a summary of participants’ stated perceptions of surplus embryos. Responses were categorized by type, and the number of mentions per category was counted. Some participants gave multiple responses.

The participants described a range of perceptions (Figure 1) that fell into three main categories: (1) life, (2) genetically related, and (3) cells or eggs. None of the participants described embryos as both “life” and “cells” or “eggs”; these categories were mutually exclusive. However, references to genetically related embryos were found for both groups.

#### 3.3.1 Perceiving embryos as life

The perception of embryos as “life” was the most frequently expressed view among participants. Some noted that this view became stronger after experiences such as childbirth and miscarriage. All participants who described embryos as life also explained that, in their view, embryos are not yet human beings. Personhood is commonly associated with specific developmental milestones such as the detection of a heartbeat via ultrasound or the recognition of a head-and-body (two-part) shape. Some participants described embryos using expressions such as “the baby coming back” or “waiting in the sky.”

#### 3.3.2 Perceiving embryos as genetically related

Some participants described embryos as “blood ties” or as genetically connected to themselves or their already-born children. Some also described embryos as being “connected by blood,” highlighting a sense of biological or familial relatedness. Several noted that because the embryos had been created at the same time as their children, they considered them siblings of the child but not their children, as they had not been implanted. Among those who referred to embryos as cells or eggs, some still emphasized genetic continuity, saying, “the DNA is there.”

#### 3.3.3 Perceiving embryos as eggs or cells

Some participants referred to embryos as “cells” or “eggs.” Among them, several stated that they did not have strong feelings or clear ideas about embryos and described them in vague or neutral terms, such as “just eggs” or “maybe just cells.” Others emphasized a scientific or legal basis for their view, saying that embryos were “not life” or “just cells that would naturally be lost without implantation.” Some participants explained that, although they sometimes felt that embryos might be more than just cells, they chose to think of them in this way to avoid emotional distress, especially after difficult treatment experiences or miscarriages.

### 3.4 Theme 2: decisions on embryo disposition

**Figure 2:**
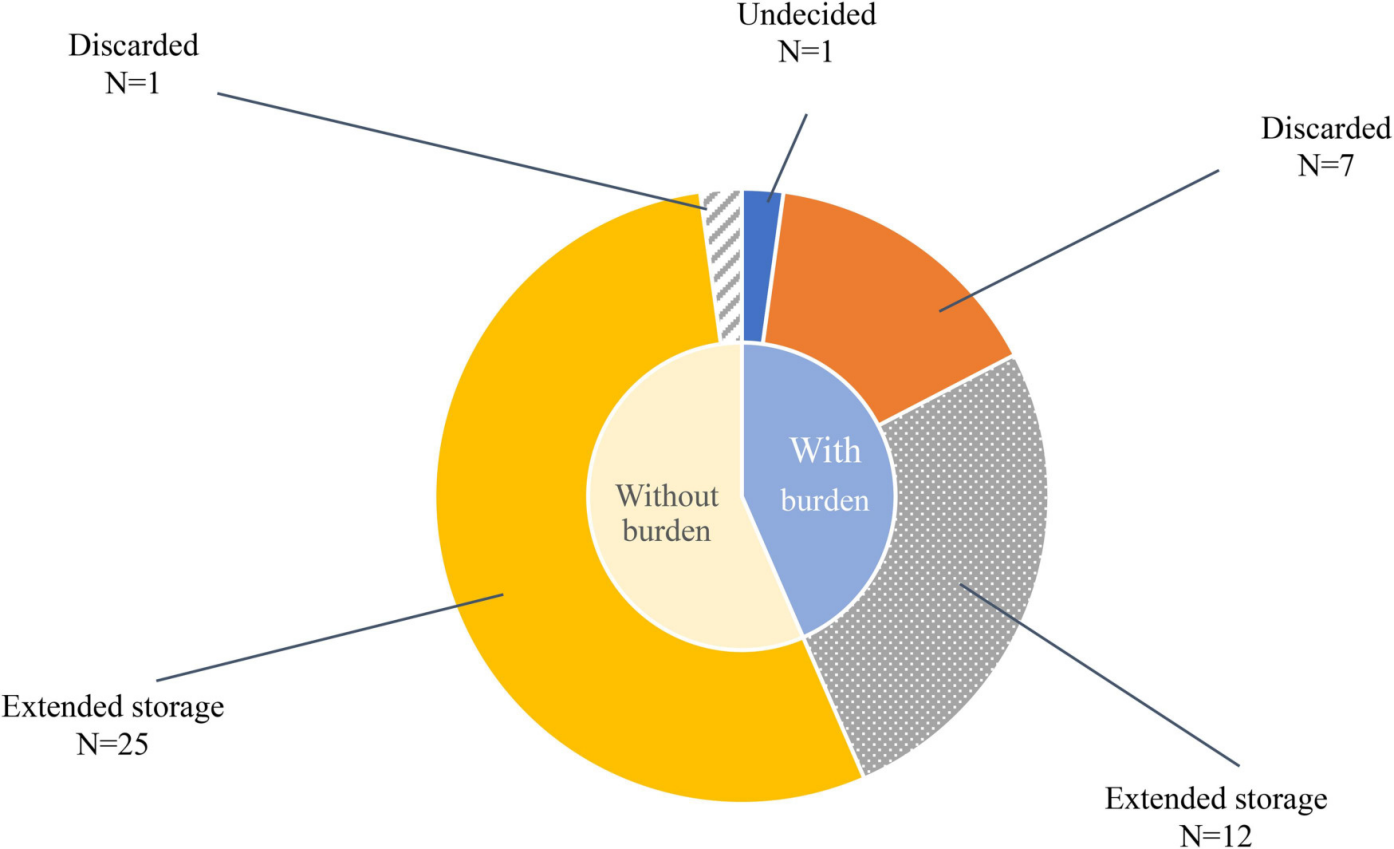
Embryo disposition choices and emotional burden. The inner circle represents whether participants experienced emotional burden (“with” vs. “without”). The outer circle shows their embryo disposition choices within each group: discarded, extended storage, or undecided. One participant remained undecided at the time of the interview.

Participants' decisions regarding surplus embryos fell into two main categories (Figure 2): discarding and extending storage. Emotional stress was reported in both groups, although the nature and intensity of the distress varied.

#### 3.4.1 Choosing to discard

Among the eight participants who chose to discard their embryos, seven described their decision as emotionally distressing. All eight patients were at the same facility, where donation for research was not offered as an option. Several participants reported giving up on having a second child because of their age, physical or emotional exhaustion, or a lack of support for household responsibilities and childcare. Others said that although they did not plan to have another child, they found it difficult to discard the embryos because they were seen as life, or because of the effort, money, and time involved in creating them. However, high storage costs have led them to choose disposal methods. Some participants expressed guilt, asking, “-Is it okay to discard life because of money? -” or “I want to spend money on the child I have now.” One participant reported no distress and explained that she and her spouse jointly decided that continued storage was unnecessary.

#### 3.4.2 Choosing to extend storage

Some participants extended embryo storage because they perceived the embryos as “precious lives” or felt that discarding them would waste the time, money, and effort spent creating them. Even without plans for additional children, they reported difficulty in deciding and describing their feelings of conflict. Some used the word *“mottainai”* —a Japanese term that express regret over waste, —to explain their hesitation. One participant said she was hurt when her spouse asked, “Then why don't we discard them?”

By contrast, participants who wished to have another child typically extended their storage without distress, regardless of the cost. Others said they were “just extending for now” because the issue had not been fully discussed or because storage fees were not a burden.

Some also mentioned the physical and emotional toll of fertility treatment, —such as pain, scheduling difficulties, and work-related disruptions —as contributing to hesitation. Few expressed the ongoing uncertainty or emotional fatigue. One remarked, “How long will I have to keep extending before I feel at ease?” Another said that she wanted to hear how others made decisions to discard their embryos.

### 3.5 Theme 3: attitudes toward embryo donation

Participants expressed their attitudes toward embryo donation, categorized as donations for research or donations to other patients.

#### 3.5.1 Donation for research

Most participants supported the donation of surplus embryos for research, particularly when faced with discarding them. Motivations included the desire to contribute to society and avoid the feeling that their treatment efforts were wasted. This supportive stance was observed across participants with varying perceptions of embryos, such —as life, genetic relationships, or cells.

However, there are several concerns. These include a lack of clear information, concerns about misuse, and the possibility of the unintended transfer of embryos. Several participants assumed that the donated embryos would only be used in infertility-related research. Some said they would consent only if the research was connected to the causes of their own infertility or the health of their children.

#### 3.5.2 Donation to other patients

Most participants expressed strong discomfort with the idea of donating embryos to other patients regardless of how they conceptualized the embryos. Those who viewed embryos as life said that they could not bear the thought of their child being raised by someone else. Participants who saw embryos as genetically related described them as on “their own” and felt uneasy about another family raising a child with their genes. Even those who referred to embryos as “cells” or “eggs” expressed resistance, saying that once implanted, the embryos would become human beings, and they would feel as if their genetic material had been taken away. One participant asked, “Can you really love a child who does not look like you?”

Only a few participants were open to this option, typically citing their empathy for others undergoing fertility treatment. However, even within this group, there was a clear preference for anonymity and no future involvement. The idea of donating to others was frequently compared with adoption, which was described as emotionally challenging or unlikely to be pursued.

### 3.6 Theme 4: views on ceremonial practices

Participants shared various views on whether symbolic or ritual practices should accompany the discontinuation of embryo storage. Notably, references to *kuyō* (a traditional Buddhist memorial rite) and other ceremonial concepts emerged spontaneously, without direct prompting. In these instances, the interviewer asked whether the participant adhered to any religious faith and all respondents were identified as non-religious. Three subthemes were identified: (1) desire for a memorial, (2) discomfort with taking the embryo home, and (3) reactions to compassionate transfer.

#### 3.6.1 Desire for a memorial

Some participants expressed a wish for a symbolic memorial despite not holding formal religious beliefs. They described emotional discomfort with embryos being treated as medical waste and some suggested that hospital-led ceremonies could help ease this burden. Others desired to take the embryos to a temple or return them to nature, for example, by scattering them at sea.

This view was observed across participants with different perceptions of embryos. Those who viewed embryos as life sometimes felt that elaborate memorials made them feel as if they had done something wrong and preferred simpler forms. Participants who referred to embryos as cells or eggs also responded positively, describing such practices as signs of compassionate care from the clinic, rather than as personal rituals.

#### 3.6.2 Discomfort with taking the embryo home

Participants were asked about “memorial disposal"—the option to receive the embryo in a preserved form, such as resin. Most participants responded negatively, stating that having an embryo at home would make them feel emotionally unsettled. Some described this idea as frightening or unnatural. One participant said, “I feel like it would haunt me.” Another said that she felt sorry about leaving it alone at home and preferred the idea of keeping it close, such as in a pendant.

While most participants reacted negatively, a few found personal meaning in the practice. One participant who viewed the embryo as life expressed a desire to be cremated with it and “meet again in heaven.” Another participant who described the embryo as a cell said that she wanted to keep it as a record of her fertility journey and show it to her child as proof of what she had been through.

#### 3.6.3 Reactions to compassionate transfer

Most participants responded negatively to the idea of compassionate transfer, which is defined as the symbolic placement of embryos in a non-receptive uterus. Those who perceived embryos as life saw them as being equivalent to intentional destruction. Others, including those who viewed embryos as cells, expressed concerns about the physical burden involved. Some participants said they would not choose it themselves but added that if someone found comfort in the practice, they would not oppose it. A few participants found the idea meaningful, with one saying, “It's not natural, but somehow it felt right.”

### 3.7 Theme 5: infertility and self-perception

Participants' understanding of the cause of their infertility and whether they identified with the label of “infertile” influenced how they perceived surplus embryos and described their emotional experiences. Three subthemes were identified.

#### 3.7.1 Impact of infertility attribution on perceptions and emotions

Participants diagnosed with male-factor infertility tended to describe embryos using instrumental terms such as “cells” or “eggs.” They often viewed fertility treatment as a practical means of achieving pregnancy and reported less emotional conflict regarding embryo disposition. One participant recalled thinking, during a discussion with her husband about treatment plans, “This is your responsibility, right?”—A reflection prompted by the context of male-factor infertility. However, she did not say it aloud. In contrast, participants with female-factor infertility or unexplained infertility more frequently described embryos as “life” and expressed more emotionally charged reactions. Some described feelings of self-blame or regret, saying things like “I’m not a normal woman” or “I wish I had acted sooner.” Those with unexplained infertility often assumed that the cause lay with themselves, especially when male factors were excluded.

#### 3.7.2 Identifying as infertile

Participants' responses to the label of “infertile” varied. Some younger participants or those who had conceived naturally in the past or had started IVF without prior steps expressed discomfort with the label. They felt that they might have conceived without assistance, or that the term placed them outside the norm.

Those with male-factor infertility often did not identify themselves as infertile because the cause was not within their bodies. In contrast, participants with female-factor infertility, —especially older women, —tended to accept the labels more readily.

A few participants said that having frozen embryos gave them a sense of reassurance, describing them as a “guaranteed chance at a next child.”

#### 3.7.3 Jealousy toward others

The participants described their experiences of jealousy at various stages, including during treatment, decisions on embryo storage or disposal, and even after childbirth. The emotional responses varied in both direction and intensity. Some participants spoke openly—almost casually—about feeling irritated or resentful toward others, such as when they saw parents with children. Although emotionally charged, these expressions are not always accompanied by signs of significant distress. In contrast, those who avoided spaces where they might encounter families (e.g., shopping malls or parks) often described a more complex and painful emotional experience. Several expressed discomfort not only with the presence of others' children, but also with their reactions, saying things like, “I still feel unsettled when others conceive easily,” or “I hated that I felt that way.”

### 3.8 Theme 6: support needs

This theme explored the participants' preferences and attitudes toward emotional support, particularly in the context of embryo disposition.

#### 3.8.1 Interest in counselling

The participants who experienced distress often expressed a desire to talk to others. Some welcomed the idea of professional counselling, while others preferred more informal or flexible formats, such as online sessions. One participant said, “With all the housework and childcare, I just can't take time to go to the hospital for counselling.”

Several participants expressed hesitation. They worried that counselling might involve judgment, offer little benefit, or be emotionally burdensome. Some were concerned that they might regret attending, or that others' opinions would influence their decisions. These views were not necessarily linked to distress levels, but rather to how participants personally or privately framed the issue.

Participants who preferred informal options often emphasized autonomy and emotional safety. While some stressed the need to prioritize support for those still undergoing treatment, others felt that patients who had completed treatment also needed space to reflect on the process.

#### 3.8.2 Reasons for interview participation

The participation in this study functioned as a form of support for many participants. Those experiencing distress said they “just needed someone to listen” or wanted to clarify their thoughts by speaking aloud. Others hoped their stories would help future patients, particularly those facing similar problems. Participants who reported little distress often described participation as an opportunity to give back rather than to seek help.

## 4 Discussion

This study revealed diverse perceptions regarding surplus embryos among Japanese patients who underwent IVF. Some viewed embryos as life or as genetically connected to themselves or their children, whereas others viewed them as cells or eggs. These perceptions often evolve through experiences such as childbirth or miscarriage and carry symbolic and spiritually significant meanings. Expressions like “waiting in the sky” or “coming back” suggest that embryos were not perceived merely as biological material, but as entities imbued with personal and relational meaning. This diversity contrasts with earlier Japanese studies in which participants tended to express more uniform views. In one study, embryos were described as children by all participants (15), while another reported that embryos were consistently perceived in terms of blood ties or spiritual connections, such as fate or bonds (16). These earlier findings were interpreted in light of the prevailing social norms at the time, including limited public recognition of infertility treatment and the tendency of participants to be full-time homemakers who made initial decisions individually before consulting their partners. These differences may reflect broader changes in the social landscape of infertility treatment in Japan. Although previous Japanese studies have reported that most patients perceived their embryos as representing “life” or “children,” our findings demonstrate a broader range of interpretations. Notably, more distanced expressions such as “just cells,” “eggs,” “something I feel nothing toward,” or metaphors like “insurance” and “a charm” were observed. These suggest that some participants viewed the embryos with a greater sense of psychological or symbolic detachment from themselves. This may reflect a diversification of attitudes and evolving perceptions of frozen embryos in the contemporary context. With the expansion of public insurance coverage for IVF by 2022, increased opportunities to access information through social media, and growing public awareness of male-factor infertility (17), treatments have become more widely accessible. These shifts may have contributed to more diverse ways of conceptualizing surplus embryos, particularly among couples who share decision-making responsibilities (18) and among individuals balancing fertility treatment with paid employment (19).

Among the various ways in which participants conceptualized embryos, perceiving them as “life” was particularly prominent and emotionally charged. These perceptions appear to be informed, at least in part, by Japan's cultural understanding of life and death. Animistic and Buddhist traditions, —such as the belief that spirits may reside in natural or created forms and that life is part of a cyclical process, —remain influential, even among those who do not identify with a specific religion (20, 21). By contrast, Christian-influenced frameworks, which tend to emphasize a linear concept of life and the afterlife, may shape different interpretations elsewhere (22). Such cultural orientations may help contextualize the symbolic and emotionally nuanced narratives expressed by the participants. This study also attempted to interpret participants' experiences in light of Japanese cultural contexts. For instance, the use of expressions such as “waiting in the sky” or the practice of placing embryos within the family altar reflects culturally embedded concepts of life and ancestry. Additionally, difficulties in joint decision-making with spouses may be understood in relation to the collectivist and family-centered norms common in Japanese society. While these themes were not universally shared, they suggest that cultural factors subtly influence patients' perceptions and moral distress. These findings highlight that such experiences are shaped not only by individual circumstances but also by the broader sociocultural environment in Japan. While discussions in Western literature often center on the moral status of embryos (23, 24), participants in this study tended to focus on the emotional, symbolic, and relational meanings of surplus embryos—, particularly among those who perceived embryos as “life.” We interpret these emotionally charged situations as a form of moral distress experienced by patients, referring to the conflict they felt when unable to act in accordance with their values due to institutional constraints or limited available options. Although applying the term to patient experiences is still uncommon and not yet standardized, we employ the term here in an interpretive sense to capture the intensity and complexity of participants' emotional struggles. As the following section shows, these perceptions often carried significant emotional weight and were closely tied to such distress. The participants' perceptions of embryos were closely linked to their experiences of moral distress. Those who viewed embryos as “life” often expressed guilt or sorrow, particularly when they lacked clear information or were not offered certain disposition options, such as donation for research. Although previous studies have reported that patients may feel overwhelmed by receiving detailed information early in the treatment process, many still emphasize the importance of timely explanations and structured counselling. In some countries, national policies mandate pre-treatment counselling (25), which supports patients in making emotionally challenging decisions (26).

Even participants who described embryos as “cells” or “eggs” expressed discomfort about discarding them. Several have used the term *mottainai,* —a culturally specific expression denoting regret over waste. While previous research has associated *mottainai* with the symbolic acceptance of infertility (16), the participants in this study more often referred to physical or financial investments. Among those who used pragmatic terms, three subgroups were identified: individuals who began treatment after the expansion of public insurance, those with academic backgrounds in science or law, and those who framed embryos in detached terms to manage their emotional burdens. These findings suggest that seemingly rational framing may coexist with complex emotional undercurrents.

Infertility diagnoses also influenced perceptions. Women with female-factor infertility often express guilt and inadequacy, reflecting internalized social expectations of motherhood (27, 28). In contrast, those with male-factor diagnoses described their experiences with greater emotional distance, possibly because of a perceived shift in responsibility. These patterns align with studies showing that stigma and coping strategies shape infertility-related distress (29, 30). Similarly, although not coded as a standalone theme, many participants described their reluctance to disclose their ART use to family members. This recurring pattern suggests an underlying tension between stigma internalization and the need to preserve emotional stability. Concealing ART use may have functioned as a coping strategy to avoid judgment, misunderstanding, or emotional strain, —particularly in contexts where reproductive expectations remain deeply embedded in the familial discourse. These observations underscore the importance of culturally informed support systems that recognize both overt and covert stigma.

Practical constraints also influence decision-making. Notably, all participants who chose to discard embryos were affiliated with clinics with higher storage fees. By contrast, those who continued storage, —even without plans for further treatment, —typically attended clinics with lower fees. Several participants noted that, while IVF itself was covered by public insurance and was thus financially manageable, storage fees remained out-of-pocket expenses, which made them feel disproportionately high. This suggests that institutional cost structures, —particularly in the context of partial insurance coverage, —may shape perceptions of fairness and contribute to passive continuation or the pressure to discard. Although this trend was consistent in the present sample, further studies are needed to assess its broader implications.

Most participants favored embryo donation for research over discarding it, although many expressed a desire to place limits on how their embryos would be used. Concerns regarding vague explanations and potential misuse are common (31, 32). In contrast, donations to other patients were met with stronger resistance, regardless of how the embryos were conceptualized. This suggests that decisions were shaped less by the ontological views of the embryo and more by cultural norms surrounding kinship, genetic continuity, and personal responsibility. Even participants who described embryos as “just cells” voiced discomfort with the idea of someone else raising a genetically related child. Conversely, those who perceived embryos as life did not necessarily want them to be born and raised outside their families. These reactions may reflect Confucian values that emphasize bloodline and family boundaries (22) as well as Japan's generally low societal acceptance of adoption (33). Although gamete donation is permitted under certain conditions in Japan, —such as anonymous donation between married couples and institutional oversight (34), —there is currently no legal framework for embryo donation. Several participants argued that embryos, as “already started life,” differ fundamentally from gametes. These findings highlight the need for a policy development that is culturally sensitive and responsive to patients' life experiences.

Support needs varied across participants. Many valued symbolic practices such as kuyō rites—not as religious obligations, but as meaningful acts of closure. In contrast, “memorial disposal” (e.g., resin-sealed embryos) and compassionate transfer (35, 36) are often viewed as emotionally burdensome or ethically troubling. These practices should not be offered as standard care and must be aligned with individual values and preferences.

Preferences for psychological support also differed. While some participants welcomed counselling, others found it too formal, intrusive, or time-consuming. Notably, those reluctant to seek counselling often spoke openly during the interviews, suggesting that informal, peer-based, or lightly facilitated spaces may be more acceptable. This finding is consistent with prior research on mental health stigma and self-reliance in Japan (37, 38).

To accommodate these diverse needs, a stepped care model may be the most effective. This could include brief psychological screening, support from trained non-specialist staff, and digital tools, such as decision aids or self-guided modules. Stepped care has been shown to enhance engagement and accessibility in other reproductive health contexts such as perinatal mental health (39). These scalable approaches could help ensure more patient-centered and culturally responsive care in Japan, —especially because the expansion of public insurance for ART is expected to increase the number of patients and strain the availability of clinical resources.

## 5 Limitations and future directions

This study had several limitations. First, as the interview guide was developed based on previous studies and refined collaboratively among the authors, it may have incorporated subjective perspectives, which constitutes a limitation of the study.

Second, only a small number of participants chose to discard embryos, which limited their insight into the emotional and ethical reasoning behind their decision. As most participants were in their 30s to 40s, had undergone a single egg retrieval cycle, and achieved pregnancy after one or two embryo transfers, the sample may not represent the full spectrum of IVF patients—particularly those in younger or older age groups—limiting the generalizability of the findings.

Third, all participants were women, and the perspectives of male partners or individuals who had disengaged from fertility care were not examined. This recruitment approach reflected the structure of fertility care in Japan, where treatment is typically centered on the female patient's medical chart. Nonetheless, several participants expressed a wish for their partners' perspectives to also be heard, underscoring the importance of including male voices in future research.

Fourth, all eight participants who had completed treatment were recruited from a facility where embryo donation for research was not offered, meaning their decisions may have been influenced by the limited options available to them—a potential option bias that should be considered when interpreting the findings. In Japan, the availability of embryo donation for research varies across fertility clinics, and not all patients are presented with this option. Although national data on clinic-level practices are lacking, such institutional differences may shape patients' decision-making and perceptions of surplus embryos.

Fifth, although feelings of jealousy toward others undergoing treatment were mentioned, they were not examined in depth. Similarly, many participants described their reluctance to disclose their ART use because of concerns about stigma, especially within families. This tendency toward secrecy, although not coded as a standalone theme, warrants further exploration in future studies.

Finally, the strong resistance observed toward embryo donation to other patients suggests the influence of sociocultural norms unique to Japan. Broader nationwide surveys and mixed-method research are needed to clarify how personal, cultural, and institutional factors intersect to shape patients' decisions and support needs.

## 6 Conclusion

This study demonstrates that Japanese IVF patients' perceptions of surplus embryos are shaped by a combination of cultural values, emotional experiences, and institutional conditions. Many participants perceived embryos as “life” or as genetically connected to themselves or their children, while others—particularly those who began treatment after the expansion of public insurance—adopted more pragmatic views, referring to embryos as “just cells” to ease emotional burden.

Although previous Japanese studies have reported that most patients perceived their embryos as representing “life” or “children,” our findings demonstrate a broader range of interpretations. Notably, more distanced expressions such as “just cells,” “eggs,” or “something I feel nothing toward” were observed, suggesting a greater sense of psychological or symbolic detachment in some participants. This may reflect a diversification of attitudes and evolving perceptions of frozen embryos in the contemporary context.

Gendered responses to infertility were evident, women with female-factor diagnoses often internalized guilt, whereas those with male-factor diagnoses described greater emotional distancing. Decisions about embryo disposition were influenced by symbolic meaning, economic considerations such as storage fees, and the policies of the clinics they attended. In addition to cultural and psychological factors, practical constraints such as storage fees also shaped patients' decisions regarding surplus embryos. These findings underscore the need to consider economic as well as cultural contexts when designing support systems.

Participants expressed diverse needs for closure and psychosocial support. Although embryo donation for research was occasionally accepted, donations to other patients were consistently rejected, highlighting the influence of cultural norms on kinship and genetic ties.

Preferences for support ranged from formal counselling to informal, peer-based dialogue. These findings call for the development of culturally sensitive policies, including clear national guidelines on embryo donation, transparency regarding embryo disposition options, and flexible, patient-centered models of care.

## Data Availability

The raw data supporting the conclusions of this article will be made available by the authors, without undue reservation.
