# Supplementary material for: Perceptions and moral distress in surplus embryo disposition among Japanese IVF patients: a qualitative study
Source: Front Reprod Health. 2025 Sep 4;7:1646340. doi: 10.3389/frph.2025.1646340 (PMC12443776; doi:10.3389/frph.2025.1646340)
Supplement: Supplementary file 1 [file Table1.docx]

Supplementary Table 1. Summary of thematic categories and frequency of participants expressing each view

| Theme | Subcategory | n / Total |
| --- | --- | --- |
| Theme 1: Perceptions of surplus embryos | Life | 25 / 46 |
|  | Connection to oneself | 19 / 46 |
|  | Connection to the born child | 12 / 46 |
|  | Egg/cell | 12 / 46 |
| Theme 2: Decisions on embryo dispositions | Discard | 8 / 46 |
|  | Gave up second child | 6 / 8 |
|  | Financial reason | 6 / 8 |
|  | Continue cryopreservation | 37 / 46 |
|  | Because important | 4 / 37 |
|  | Because wasteful | 4 / 37 |
|  | For the time being | 7 / 37 |
| Theme 3: Attitudes Toward Embryo Donation | To research – agree | 36 / 46 |
|  | To research – disagree | 6 / 46 |
|  | To others – agree | 8 / 46 |
|  | To others – disagree | 23 / 46 |
| Theme 4: Views on Ceremonial  Practices | Desire for a Memorial | 8 / 46 |
|  | Taking the embryo home – agree | 9 / 46 |
|  | Taking the embryo home – disagree | 28 / 46 |
|  | Compassionate Transfer – agree | 4 / 46 |
|  | Compassionate Transfer – disagree | 12 / 46 |
| Theme 5: Infertility and  Self-Perception | Male factor | 8 / 46 |
|  | Female factor | 14 / 46 |
|  | Unexplained | 23 / 46 |
|  | Perceives self as infertile | 8 / 46 |
|  | Does not perceive self as infertile | 14 / 46 |
|  | Jealousy toward others | 5 / 46 |
| Theme 6: Support needs | Interested in counseling | 3 / 46 |
|  | Not interested in counseling | 13 / 46 |
|  | Interview motive – to be heard | 6 / 46 |
|  | Interview motive – to help others | 7 / 46 |

Note: Participants could express more than one view within each theme; therefore, totals may exceed the number of participants.
